# Chronic Exposure of Corals to Fine Sediments: Lethal and Sub-Lethal Impacts

**DOI:** 10.1371/journal.pone.0037795

**Published:** 2012-05-25

**Authors:** Florita Flores, Mia O. Hoogenboom, Luke D. Smith, Timothy F. Cooper, David Abrego, Andrew P. Negri

**Affiliations:** 1 Australian Institute of Marine Science, Townsville, Queensland, Australia; 2 Australian Research Centre of Excellence for Coral Reef Studies, James Cook University, Townsville, Queensland, Australia; 3 Australian Institute of Marine Science, University of Western Australia Oceans Institute, Western Australia, Australia; Smithsonian’s National Zoological Park, United States of America

## Abstract

Understanding the sedimentation and turbidity thresholds for corals is critical in assessing the potential impacts of dredging projects in tropical marine systems. In this study, we exposed two species of coral sampled from offshore locations to six levels of total suspended solids (TSS) for 16 weeks in the laboratory, including a 4 week recovery period. Dose-response relationships were developed to quantify the lethal and sub-lethal thresholds of sedimentation and turbidity for the corals. The sediment treatments affected the horizontal foliaceous species (*Montipora aequituberculata*) more than the upright branching species (*Acropora millepora*). The lowest sediment treatments that caused full colony mortality were 30 mg l^−1^ TSS (25 mg cm^−2^ day^−1^) for *M. aequituberculata* and 100 mg l^−1^ TSS (83 mg cm^−2^ day^−1^) for *A. millepora* after 12 weeks. Coral mortality generally took longer than 4 weeks and was closely related to sediment accumulation on the surface of the corals. While measurements of damage to photosystem II in the symbionts and reductions in lipid content and growth indicated sub-lethal responses in surviving corals, the most reliable predictor of coral mortality in this experiment was long-term sediment accumulation on coral tissue.

## Introduction

Dredging for the development and maintenance of ports and harbours is becoming increasingly regulated due to the need to balance economic benefit with the principles of environmentally sustainable development. There are several dredging techniques but all cause periods of increased sedimentation and turbidity. The severity, duration and scale of impacts vary widely among techniques, and are also dependent on the depth and geological nature of the sea floor along with the hydrodynamic conditions of the area [1]. The effects of sedimentation (sediment deposition) and turbidity (elevated particulate matter in suspension and shading) on sessile benthic organisms, like corals, will therefore be critically dependent on characteristics of the site and associated dredged material, dredging technique and duration of the operations [2]. Models that predict the extent and severity of turbidity and sediment deposition and incorporate thresholds for organism health are increasingly being used as tools in the impact assessment process [3]. However, the usefulness of any model prediction hinges on robust inputs on the biological response to the environmental stressor in the receptor organism of interest.

Sedimentation is defined as the deposition of particulate material onto the benthos, with the origin of the particles as resuspension from the seafloor or new imports through terrestrial runoff [2], [4] or dredging [5], [6]. Sediment deposition rates in tropical marine habitats are highly variable and rates of 300 mg cm^−2^ day^−1^ are not unusual [7], [8]. Exposure to sediments can produce a range of different responses in corals. For example, feeding on fine sediment particles may enhance coral growth in some species [9] although the potential nutritional gain from feeding on particulate organic matter is species-specific and depends on the sediment-type [10]. In general, however, settling of particulate matter onto the colony surface is considered a stress to corals because sediment rejection leads to down-regulation of photosynthesis and increased rates of respiration and mucous production [11]–[14]. Photo-physiological stress occurs within hours of exposure to sedimentation [13], [15] and is strongly related to grain size, organic content and nutrient composition of the sediment [15], and has been considered a useful sub-lethal bioindicator of changes in water quality [16]. With increasing exposure to sediments, coral growth rates decline, symbionts are known to be expelled (bleaching), and tissue loss occurs [5], [17]–[20]. Sedimentation also negatively affects rates of gamete fertilization [21] and survival and settlement of coral larvae [22], [23]. In the longer term, elevated sedimentation regimes can influence coral cover and community composition due to differences in sediment tolerances among species [24].

Turbidity is a measure of the amount of suspended particulate matter (SPM) in the water column (and to a lesser extent some dissolved organic compounds) and their effect on light attenuation [25]. Both organic (bacteria, phytoplankton, zooplankton and detritus), or inorganic (sediment) particles contribute to the SPM and total suspended solids (TSS) can reach 300 mg l^−1^ during dredging operations [2], [6], [26]. Turbidity and light attenuation can vary over small spatial and temporal scales depending on the proximity of sources of terrestrial runoff [27] as well as changes in local weather conditions [4], [28], [29]. Turbidity and light attenuation can have contrasting effects on corals. Some species gain a substantial proportion of their energy budgets from heterotrophic feeding on SPM, while others obtain most of their nutrition from autotrophy (symbiotic zooxanthellae provide the host coral with sugars, amino acids, lipids and peptides [30]) regardless of the availability of particulate matter [10]. In deep water, energy lost from reduced light availability may be offset by the energy gained from utilizing SPM [27], [31]. To cope with variation in light levels, corals are able to photo-acclimate by adjusting the concentration of photosynthetic pigments and/or the density of their symbionts. Under low irradiance, corals may exhibit higher concentrations of photosynthetic pigments and/or symbiont densities [31]–[34]. Corals that are not able to compensate energetically for reduced light availability may experience decreased rates of calcification and thinner tissue in the coral host [14], [35], [36]. Corals in this reduced energy state may be more vulnerable to thermal bleaching, but alternatively can be protected from bleaching as turbidity due to increased SPM can reduce harmful irradiance [37].

The levels of sedimentation and turbidity that impact on corals vary according to species, polyp size and growth form [38]. In general, corals are thought to be affected by chronic sediment deposition rates greater than 10 mg cm^−2^ day^−1^ and TSS above 10 mg l^−1^ [2], but this is highly dependent on sediment properties (corals have the greatest difficulty in expelling and removing the finest sediment fractions) [15]. While field studies on benthic habitats near dredging operations clearly demonstrate effects on individual organisms and ecosystem structure [2], [5], [6], specific mortality thresholds and direct cause and effect relationships are difficult to assign due to variability within, and interactions among, shading, TSS and sediment deposition rates. Laboratory studies that control light intensity, TSS, sediment deposition rates and temperature are more suited to quantifying the specific effects of various environmental changes associated with dredging activities. However, great care in experimental design must be taken to ensure environmental relevance. The sediment particle size, the duration of the exposures, adequate flow-through conditions and sediment contamination analyses are some of the critical factors that need to be carefully chosen at the start of each experiment [39]. The selection of suitable study species is also critical to represent a range of expected sensitivities to sedimentation, TSS and shading. A well-planned and controlled experimental exposure of corals to sediments can provide management with mortality and sub-lethal thresholds for individual stressors (e.g. TSS or sediment deposition rates); evidence for cause and effect relationships that may be observed in the field; the opportunity to examine the interaction of multiple stressors under controlled conditions; and finally, controlled experiments offer the potential to develop and/or validate new bioindicators of coral stress for field deployment. To date, there has not been any published experimental data that quantifies sedimentation and turbidity thresholds for offshore corals, which are not commonly exposed to terrigenous particulates and may have different response thresholds than nearshore corals.

**Figure 1:**
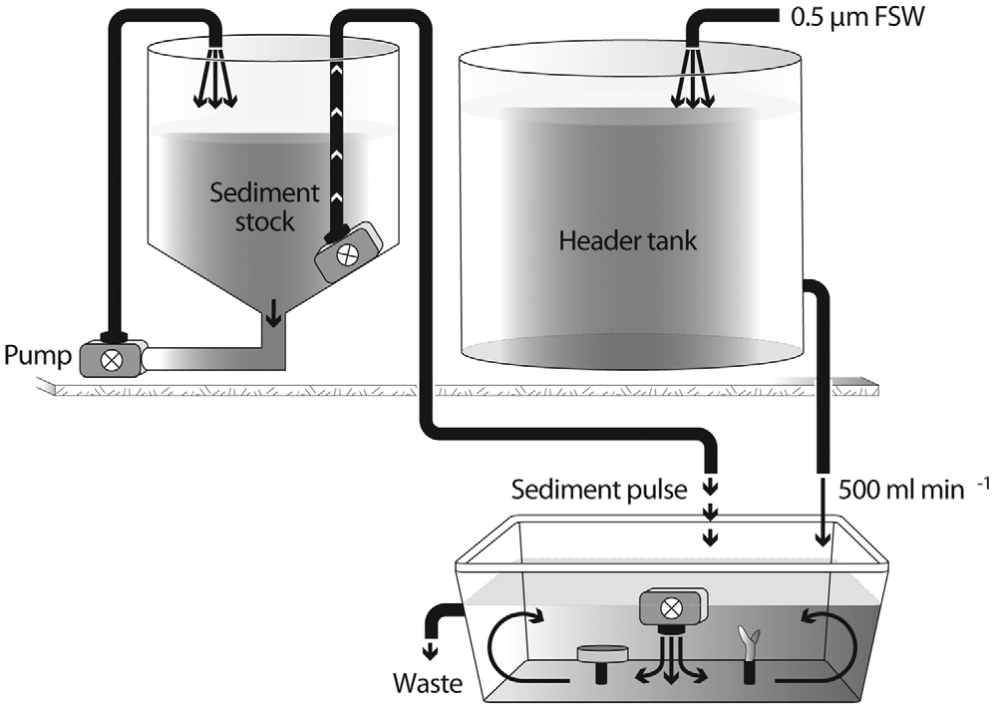
Diagrammatic representation of the flow-through dosing system.

**Figure 2:**
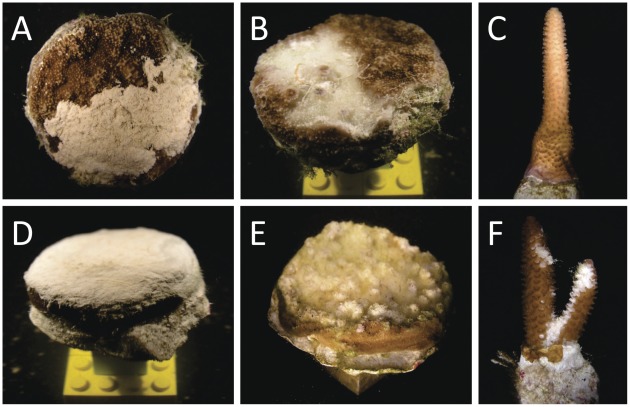
Representative examples of *M. aequituberculata* (A, B, D & E) and *A. millepora* (C & F) corals in (A–C) 30 mg l ^−**1**^ **and (D–F) 100 mg l** ^−**1**^ **TSS treatments after 12 weeks.** Note that (A) partial sediment cover led to partial mortality (B) and (D) full sediment cover led to (E) full mortality. However, note also small areas of live tissue on the vertical sides of (E) the 100 mg l^−1^ TSS *M. aequituberculata*.

The present study used a laboratory-based, experimental approach to examine the responses of corals to chronic sedimentation in order to develop lethal and sub-lethal thresholds for corals exposed to dredging-generated sediments relevant to offshore developments adjacent to coral reefs. Very fine sediments were generated from coral sand to a particle size consistent with material that can be found up to 500 m from dredging activities. The corals were exposed to reef-type sediment in a custom designed flow-through aquarium (Fig. 1). The corals used were fragments from two common Indo-Pacific species representing branching and foliaceous morphologies (Fig. 2). The two primary objectives for the study were: (1) to measure the sub-lethal health indicators and overall mortality of corals exposed to a range of turbidity and sedimentation levels over a long-term exposure (12 weeks); and (2) study the potential recovery, using the same sub-lethal indicators for an additional 4 weeks after the cessation of sediment exposure.

## Results

### Environmental Conditions in Tanks

The daily measured TSS concentrations were stable over the 12 week sediment exposure period and the mean measured TSS over this time was very close to the target for each treatment (Table 1, Fig. S1). Corals in control treatments (0 mg l^−1^ TSS) received 194±3 µmol photons m^−2^s^−1^ over a 12∶12 h light:dark cycle (at the level of the corals in the tanks). Only corals in the highest TSS treatment were shaded significantly (by 23% compared with the control treatment, ANOVA, F_(5,219)_ = 24.70, p<0.01; Table 1; Fig. S2). The sediment deposition rate (SR) in the highest sediment exposure treatment was 82.7±6.2 mg cm^−2^day^−1^ (mean ± SE) over the course of the experiment (Table 1; Fig. S3 Fig. S3). Sediment deposition was consistent for each of the treatments over the course of the 12 week exposure period (Table 1 & Fig. S1).

**Table 1:** Summary of target and mean (± SE) total suspended solids (TSS), turbidity (NTU), light attenuation and sediment deposition rates in each of the experimental treatments.

Target TSS (mg l^−1^)	MeasuredTSS (mg l^−1^)	Turbidity(NTU)	Light attenuation(% rel. to 0 mg l^−1^)	Sediment deposition rate(mg cm^−2^day^−1^)
0	0.19 (0.02)	0.10 (0.01)	^−^	0.43 (0.04)
1	1.31 (0.04)	1.09 (0.04)	3.6 (2.8)	1.62 (0.16)
3	3.22 (0.07)	2.87 (0.07)	1.6 (1.3)	2.76 (0.22)
10	10.8 (0.2)	9.22 (0.18)	2.7 (1.4)	8.93 (0.56)
30	29.1 (0.5)	25.0 (0.3)	7.8 (0.6)	25.0 (2.1)
100	98.2 (1.9)	83.6 (1.0)	23.4 (1.8)	82.70 (6.2)

### Mortality and Sediment Accumulation

**Figure 3:**
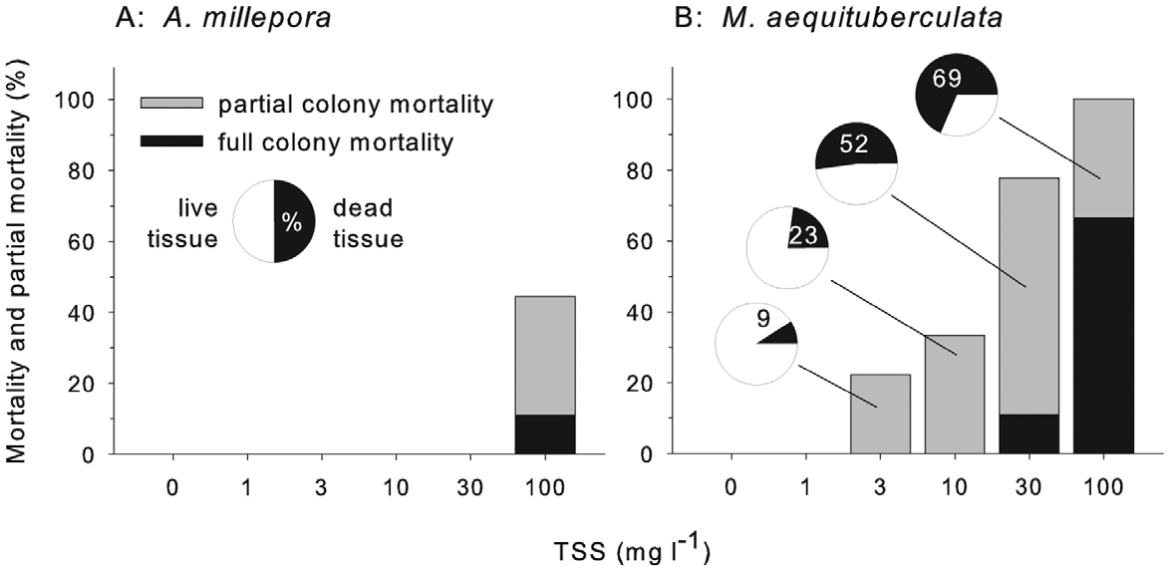
Whole and partial mortality of both species after 12 weeks (n = 9). The black bars represent whole colony mortality and the grey bars partial mortality. The proportion of the surface area of *M. aequituberculata* (mean %) that was dead in partial mortality colonies is represented by black slices in pie charts.

All coral fragments in the control and 1–10 mg l^−1^ treatments survived both the 12 week sediment exposure and subsequent 4 week recovery period (Fig. 3). In the two highest exposure treatments, the sediment deposition rates overwhelmed the ability of *M. aequituberculata* to remove particles and heavy sediment accumulation on the horizontal surface of the colonies was observed (Fig. 2A & D), with sediment layers reaching a thickness of 4 to 7 mm after 12 weeks (Fig. 2D). Partial sediment cover of *M. aequituberculata* was recorded after 4 weeks exposure in 10, 30 and 100 mg l^−1^ (TSS) treatments, and some tissue loss was observed beneath the sediment (see below). After 12 weeks, all of the coral tissues underneath the accumulated sediments were dead, exposing white coral skeleton, often tinged with a green color due to endolithic green algae (Fig. 2B & E).

All fragments of *A. millepora* survived the 30 mg l^−1^ treatment over the 12 week exposure while 11% (1of 9) of *M. aequituberculata* fragments died at that exposure (Fig. 3). In the highest sediment exposure (100 mg l^−1^, 82.7 mg cm^−2^ day^−1^) 67% of the 9 *M. aequituberculata* fragments were completely dead by week 12 (Fig. 3B). The number of dead fragments did not increase during the 4 week recovery period. Sediments did not accumulate on *A. millepora* apart from over small areas <1 cm^2^ (Fig. 2C). Small patches of tissue mortality in *A. millepora* resulted from these minor sediment accumulation patches (Fig. 2F). Consequently, over the 12 week exposure period to 100 mg l^−1^ TSS (82.7 mg cm^−2^ day^−1^), only one *A. millepora* fragment (11%) had died (Fig. 3A).

Partial mortality, characterized by severe tissue loss and bare skeleton, was only observed for 33% (3 of the 9) of *A. millepora* fragments exposed to 100 mg l^−1^ TSS (82.7 mg cm^−2^ day^−1^) at 12 weeks (Fig. 3A). The surface area of tissue affected was less than 10% but these 3 corals were dead at the end of the subsequent 4 week recovery period. Partial mortality of *M. aequituberculata* fragments was detected in the 3 mg l^−1^ TSS (2.8 mg cm^−2^ day^−1^) treatments; however, only an average of 9% of the surface was dead in these two corals (Fig. 3B). The proportion of the surface of *M. aequituberculata* exhibiting tissue death increased to 52% of 6 corals at 30 mg l^−1^ TSS (25 mg cm^−2^ day^−1^) and 69% of the 3 remaining live corals exposed to 100 mg l^−1^ TSS (82.7 mg cm^−2^ day^−1^) by week 12 (Fig. 3B).

**Figure 4:**
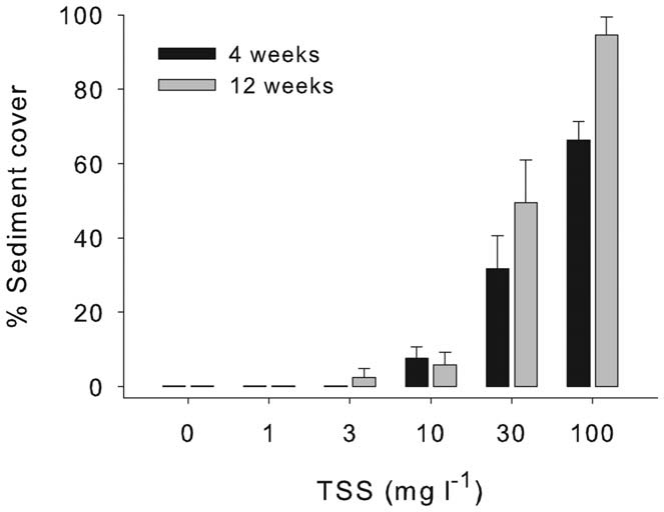
Mean sediment cover on the surface of *M. aequituberculata* following 4 and 12 weeks of exposure to sediments (+ SE, n = 9).

Sediment accumulation on the surface of *M. aequituberculata* was evident at 10 mg l^−1^ TSS (8.9 mg cm^−2^ day^−1^) after 4 weeks and at 3 mg l^−1^ TSS (2.8 mg cm^−2^ day^−1^) after 12 weeks (Fig. 4). The area of sediment accumulation on the surface was influenced by both sediment deposition rates and time (Table S1; F_(3,35)_ = 23.71, p<0.000, F_(1,35)_ = 10.95, p = 0.002 ). In the highest sediment treatment (100 mg l^−1^, 82.7 mg cm^−2^ day^−1^), 66±5% and 95±5% (SE) of the horizontal surfaces of *M. aequituberculata* were covered by sediments after 4 and 12 weeks, respectively (Fig. 4).

**Figure 5:**
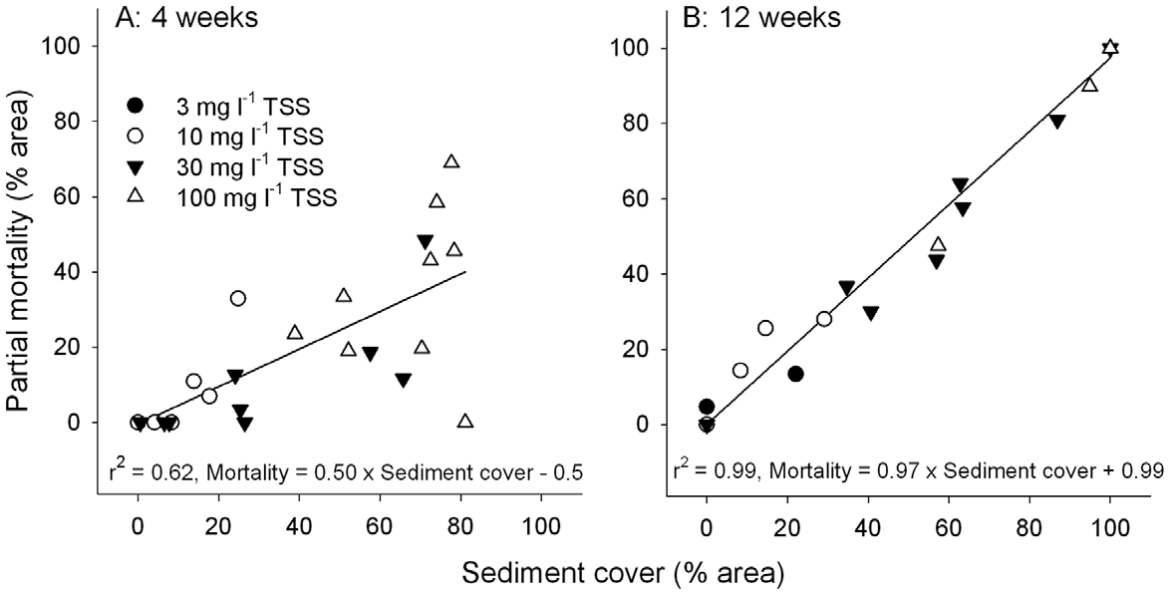
Regressions of sediment cover (%) against partial mortality (%) of the surface tissues *M. aequituberculata* at (A) 4 and (B) 12 weeks (n  = 9).

Linear regressions revealed a strong relationship between sediment cover on the upper horizontal surface of *M. aequituberculata* and the extent of partial tissue mortality (Fig. 5). After 4 weeks, half of the area buried by sediments were dead [Mortality (%)  = 0.50×Sediment cover (%) –0.5, r^2^ = 0.62] while almost all of the tissue covered by sediments were dead after 12 weeks [Mortality (%)  = 0.97×Sediment cover (%) +0.99, r^2^ = 0.99].

### Biochemical and Physiological Indicators

Sediment treatments significantly affected the growth of *A. millepora* (Fig. S4; F_(5,96)_  = 5.31, p<0.001). An increase in growth rate in corals exposed to moderate sediment treatments (10 mg l^−1^) is potentially related to heterotrophic feeding. However, growth rates in corals exposed to the highest sediment treatment (100 mg l^−1^ TSS) were significantly depressed (but still positive) (Fig. S4).

**Figure 6:**
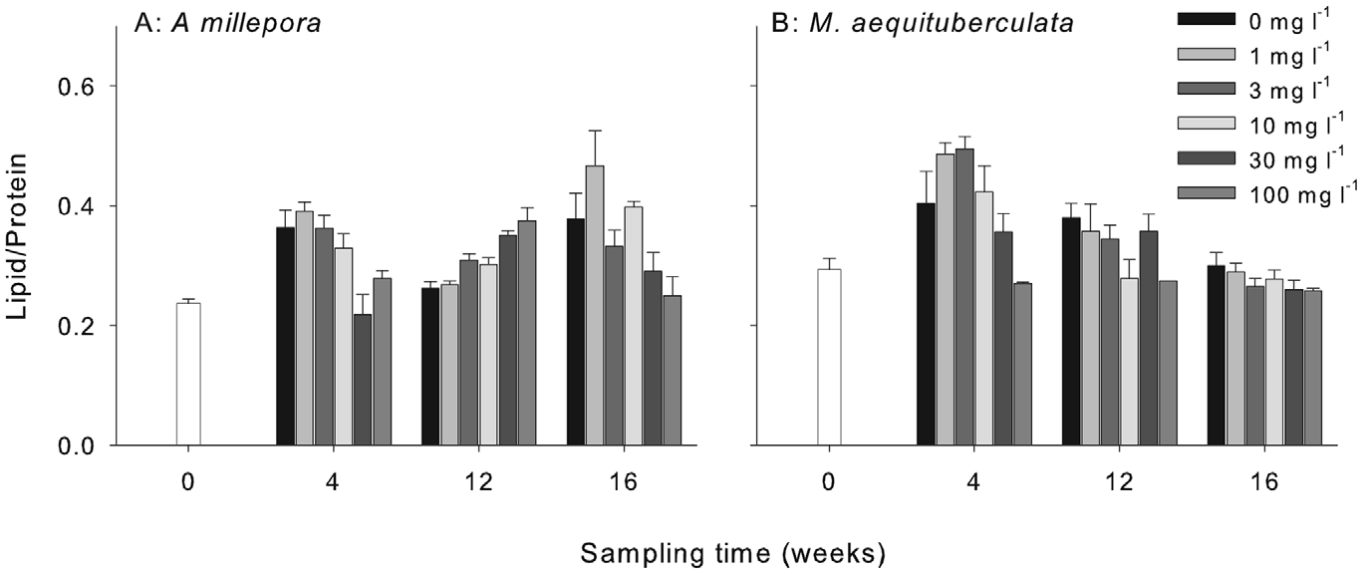
The effect of sediment treatment on lipid normalized to biomass (mg lipid/mg protein) at each of the sampling periods for (A) *A. millepora* and (B) *M. aequituberculata* (bars + SE, n = 9 corals per treatment per sampling period). Nine corals were collected at t = 0 days to represent all of the initial treatments.

Lipid content was used as a proxy for stored energy in the coral tissue. Significant reductions in lipid content compared with controls were observed for *M. aequituberculata* following sediment exposures (Fig. 6B, Table S2). In this species lipid content was lower for the highest sediment treatment after both 4 and 12 weeks (reduced by 32% and 28%, respectively, relative to controls) and generally decreased over time. The highest lipid content was observed in the 3 mg l^−1^ TSS after 4 weeks (22% higher than controls). Following the recovery period, surviving corals exhibited lipid content more consistent with corals at the start of the experiment (less than 12% difference) (Fig. 6B). There was no consistent or statistically significant influence of sediment treatment on lipid content in *A. millepora* (Fig. 6A, Table S2).

**Figure 7:**
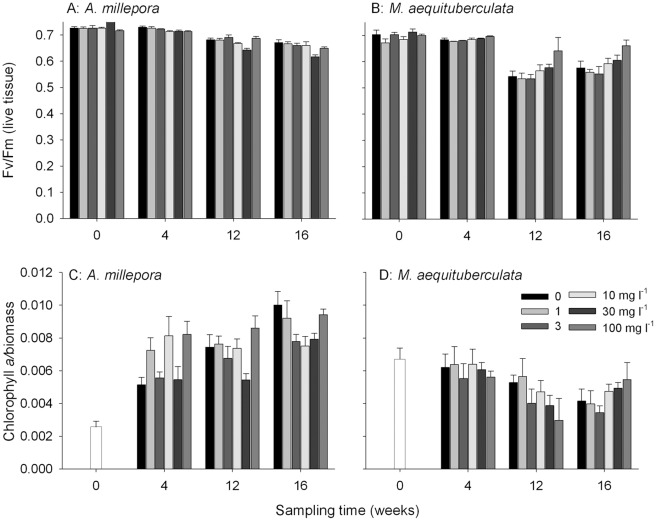
Mean maximum potential quantum yields (F_v_/F_m_) (+ SE, n = 9) for (A) *A. millepora* and (B) *M. aequituberculata* for each of the sediment treatments over the 16 week experiment. Chlorophyll *a* concentration normalized to biomass at each of the sampling periods for (C) *A. millepora* and (D) *M. aequituberculata* (bars + SE, n = 9 corals per treatment per sampling period). Nine corals were collected at t = 0 days to represent all of the initial treatments.

A decrease in maximum quantum yield (F_v_/F_m_, an estimate of photosynthetic efficiency in Photosystem II) was observed for both species with time and this reduction continued into the recovery period for *A. millepora* (Fig. 7A, B; Table S3). There was no effect of sediment treatment on F_v_/F_m_ over the 12 week exposure period in *A. millepora* (Table S3); however, a significant decrease in F_v_/F_m_ in the two highest sediment treatments was observed following the recovery period in this species (reduced by a maximum of 8% in 30 mg l^−1^ TSS treatments compared to controls). The reverse trend was observed for *M. aequituberculata*, which exhibited higher F_v_/F_m_ values at 30 mg l^−1^ and 100 mg l^−1^ TSS (25 and 82.7 mg cm^−2^ day^−1^, respectively; Fig. 7B). F_v_/F_m_ was 18% higher than controls in *M. aequituberculata* after 12 weeks exposure to the highest sediment treatment and a similar result was observed following the recovery period.

Chlorophyll (chl) *a* concentrations were used to quantify potential bleaching (reduced symbiont density) in sediment-exposed corals. The concentration of chl *a* in *A. millepora* was significantly affected by sediment treatment (Table S4)) and chl *a* increased in *A. millepora* over both the exposure period and during recovery, reaching approximately 4 times the initial concentration (Fig. 7C) by week 16. There was no significant difference in chl *a* content of *M. aequituberculata* colonies after 4 weeks or 12 weeks (Table S4), although chl *a* in corals exposed to the highest sediment treatment contained 43% less chl *a* than the control corals (Fig. 7D) after 12 weeks. Chl *a* concentration after recovery was lower than the initial concentration though the chl *a* in corals exposed to the highest sediment treatment was higher than chl *a* in controls at that time point.

## Discussion

This was the first laboratory study on the chronic effects of sedimentation and turbidity on coral sampled from offshore environments. Lethal and sub-lethal sedimentation/turbidity thresholds were identified with *Montipora aequituberculata* proving more sensitive to sediment treatments than *Acropora millepora*. While little sediment accumulation was observed for branching *A. millepora,* sediments readily accumulated on the foliaceous *M. aequituberculata* in concert with increased sedimentation. The high correlation between the extent of tissue mortality and sediment deposition on the surface of *M. aequituberculata* indicated that sediment deposition rather than elevated TSS was the primary cause of stress in this species. While most of the biochemical and physiological measures indicated sub-lethal stress in corals or their symbionts due to the elevated sediments, this was usually only evident in the highest two sediment treatments. Sediment accumulation on coral tissue was a strong and consistent cause (and predictor) of tissue mortality, and resulted in mortality of whole coral fragments over prolonged periods.

Sediment deposition onto coral tissue can cause mortality in corals by: (i) suffocation of tissue under anoxic conditions [2] and this can be influenced by nutrient composition and microbial activity [15] and (ii) starvation through depression of photosynthesis or heterotrophic feeding [2]. Corals have a variety of mechanisms to actively remove sediment particles, including the use of their cilia and tentacles, distension of coral polyps with water to form waves and increase gas exchange [13] and mucous production to capture particles for later removal [2], [40]. In the present study, prolonged and/or intense sediment deposition is likely to have overcome capacity of the coral (or the additional energy output required) to clear sediments, which then collected on surfaces causing suffocation and anoxia. *A. millepora* was able to tolerate higher sediment deposition rates with partial and full colony mortality observed in corals exposed to 83 mg cm^−2^ day^−1^ (100 mg l^−1^ TSS) while partial mortality of *M. aequituberculata* was already observed at 2.8 mg cm^−2^ day^−1^ (3 mg l^−1^ TSS) after 12 weeks. Higher levels of mortality in *M. aequituberculata* were likely due to the larger horizontal surface area of the foliaceous species where sediments were more likely to accumulate as opposed to the vertical branches of *A. millepora*. *M. aequituberculata* also have smaller corallites and tentacles than *A. millepora,* which could have contributed to the difficulty in actively rejecting particles. This result is consistent with other studies that have compared the effects of growth form and coralite size on particle rejection [5], [18], 40,41. The TSS and sedimentation thresholds were similar to those reported for sensitive species in previous studies [2], [27], [42]. However, direct comparisons between studies is often difficult since sediment type, exposure duration, exposure type (continuous vs pulsed) and the way sedimentation is measured [15], [43], [44] will influence the reported sediment impact thresholds on corals.

In the present study, coral health was first assessed after 4 weeks and at this time, partial mortality (tissue loss) was observed in *M. aequituberculata*. The correlation between sediment cover on *M. aequituberculata* and partial mortality was significant at 4 weeks and half the tissue covered by sediments showed signs of mortality. Although impacts on coral tissue are likely to have occurred earlier than 4 weeks in the high intensity treatments (Philipp and Fabricius [13] demonstrated that tissue loss can occur in corals covered by sediments within 24–36 hours), *M. aequituberculata* may have the potential to survive heavy sediment deposition for 4 weeks. However, almost complete mortality (97%) of buried tissue after 12 weeks signifies limits to the capacity of this species to maintain viability of buried tissue over prolonged periods. These results are consistent with other studies which show that colony mortality increases with duration of tissue burial [13], [42], [45], but different coral species, sediment types and sampling methods make direct comparison between the studies difficult. Despite tissue mortality on the horizontal surfaces of *M. aequituberculata*, growth was observed on the vertical sides of the coral colonies where sediments could not accumulate (Fig. 2E). In the long term, this vertical growth may represent a mechanism to overcome, or recover from, chronic sediment deposition and, indeed, colonies of *M. aequituberculata* can exhibit limited vertical extension in the field. While some shading was observed in the highest treatment, corals in this treatment were exposed to 150 µmol photons m^−2^ s^−1^ for 12 hours daily and growth was observed for both species. In combination, the observed mortality of horizontal tissue, the high correlation between tissue mortality and sediment cover, the vertical growth of *M. aequituberculata* and low mortality in upright *A. millepora* branches indicate that sediment deposition (rather than elevated TSS) was the key pressure faced by corals in this experiment.

While smothering of coral tissue by sediments has negative impacts on the health of coral, many species can benefit from the nutrition contained in suspended solids [9]. Inshore corals exposed to high turbidity often contain higher concentrations of lipid, most likely due to heterotrophic feeding [37]. Anthony and Fabricius [10] found maximum rates of energy investment into growth at low (<10 mg l^−1^) TSS concentrations when organic carbon content of the particles was 3% w/w. In the present study the offshore sediments contained little organic carbon (0.11% w/w) and the particle size was 1–2 orders of magnitude smaller than spacing between coral tentacles (>500 µm), making active capture unlikely. There was little evidence that *A. millepora* benefited from elevated TSS since growth was only marginally increased at 10 mg l^−1^ and lipid content exhibited no consistent pattern with treatment. Although lipid content was higher in *M. aequituberculata* exposed to 1 and 3 mg l^−1^ TSS after 4 weeks, this rise was only temporary and generally decreased with increasing sediment treatment by 12 weeks. Reduced growth in *A. millepora* and reduced lipid in living *M. aequituberculata* tissue in the highest sediment treatments are most likely due to reduced energy acquisition, combined with greater energy expenditure as respiration increases during attempted particle rejection [11], [14], [35], [40]. Sediment deposition also decreases feeding efficiency, which can contribute to a decrease in lipids. The synthesis of lipids in corals is strongly correlated with light intensity [10] and shading at higher turbidity levels may have contributed to reduced lipid production by symbiotic zooxanthellae. As light decreases with higher turbidity levels corals may resort to lipid consumption as energy reserves dwindle [46].

Symbionts in both species responded differently to increased sediment treatments. Reductions in F_v_/F_m_ for *A. millepora* compared to controls at 12 weeks may indicate damage to photosystem II and this may be caused by increased TSS since there was little deposition of sediments onto the tissue of this species. The mechanism for reduced F_v_/F_m_ is unknown but may be related to a changed physiology of the host as the continuous particle rejection consumes resources usually allocated for cellular maintenance. Reduced photosynthesis:respiration ratios have also been described in corals under elevated turbidity conditions and may be due to increased respiration [14] or reduced photosynthesis [10]. The significant drops in F_v_/F_m_ for *M. aequituberculata* at 12 and 16 weeks may be due to cumulative photo-damage to PSII (due to oxidative stress) or cumulative down-regulation of PSII photochemical efficiency (an adaptation to higher light) [47]. This is likely if the light intensity of the experimental conditions exceeded the intensity from where the corals were collected (this is unknown). The increase in F_v_/F_m_ in *M. aequituberculata* exposed to high sediments (at 12 weeks, relative to controls) could result from low-light adaptation due to the minor shading caused by elevated TSS (symbionts adapt to increase photosynthetic efficiency in more turbid conditions). Endolithic algae were frequently observed growing within the coral skeleton that had been buried by sediment for 12 weeks. While only data on live tissue are presented here, care must be taken to avoid the possibility that endolithic algae may contribute to the fluorescence signal when using changes in photosynthetic yield as an indicator of coral health.

Chlorophyll *a* concentrations were significantly correlated with symbiont density (see Materials and Methods) and used as a measure of bleaching in the sediment-exposed corals. Since tissue damage and partial mortality were observed on many of the corals in higher treatments chl *a* was normalized to biomass (protein content). This provided a measure of pigmentation relative to live tissue. There was a seemingly large reduction in chl *a* content due to sediment exposure for *M. aequituberculata* (at 12 weeks). This potential reduction of 43% compared to controls (Fig. 7D) was not statistically significant but any genuine decrease may have been due to host stress leading to expulsion of symbionts. Loss of symbionts has been reported previously in corals affected by sedimentation [11], [13], [35], [41] but any subtle effects of sediment exposure on symbiont density or pigment concentration is likely to have been masked by the long exposures used here. There was an increase in chl *a* in *M. aequituberculata* exposed to the highest sediment treatment following the 4-week recovery but the presence of green endolithic alga on some of those coral fragments may have contributed to this increase. The increase in chl *a* observed for *A. millepora* over the course of the experiment (Fig. 7C) is likely due to an increase in the density of symbionts in this species as the corals slowly acclimated to the light environment used in the experiment.

The effect of sediment exposure duration was significant for all indicators of sub-lethal stress (reduced lipid, growth, F_v_/F_m_ and/or chl *a*) in both species. In some cases there were significant interactions between treatment intensity and time. For example, there was a significant decline in F_v_/F_m_ over time for *A. millepora* while the effect of exposure intensity on F_v_/F_m_ was inconsistent at each time point. More time points would be needed to explore the nature of most of those interactions but this is usually impractical for measures such as destructive lipid and chl *a* measurements. There was little opportunity to explore potential for recovery over the subsequent 4 week period. During this time further mortality in *A. millepora* was observed following exposure in the highest sediment while no further mortality was observed in *M. aequituberculata.* Longer recovery periods, coupled with realistic sediment pulses and shading (to mimic light reduction caused by increased TSS over a longer light path [48]) are required in future experiments to better estimate the resilience of corals to sediment exposure. The large differences in sensitivity of the two corals here to sediment deposition also highlights the need to broaden the scope of species tested and to further explore the potential effects of dredging on the vulnerable early life stages of coral [21]–[23].

To adequately manage the impacts of dredging in marine systems requires the ability to confidently predict the spatial extent and severity of likely impacts. Predictive models require robust quantitative inputs including sediment pressure thresholds for organism mortality and sub-lethal effects [3], [49], [50]. While well conducted field studies are critical to inform this process [5], [6], [48], laboratory-based experiments can also deliver valuable, quantitative inputs to impact assessment models. This experiment successfully delivered consistent TSS and sediment deposition rates over prolonged periods, using sediments that are likely to be generated in dredging programs adjacent to coral reefs. Threshold concentrations for lethal and sub-lethal stress in corals were derived for two species. Full colony mortality in some colonies was observed at 30 mg l^−1^ TSS (25 mg cm^−2^ day^−1^) for *M. aequituberculata* and at 100 mg l^−1^ TSS (83 mg cm^−2^ day^−1^) for *A. millepora* after 12 weeks. While common branching coral species are likely to survive prolonged periods of high sedimentation (up to 83 mg cm^−2^ day^−1^ or 100 mg l^−1^ TSS), corals with horizontal growth forms are particularly vulnerable to ultra fine sediments, which would be expected to travel further from dredging operations due to their relatively low settlement velocity [50]. Intense sediment treatments generally had a negative impact on both species of coral; however, the increased growth and lipid content observed in some of the low sediment treatments indicates possible nutritional benefits to corals and both outcomes are likely following dredging events. With the rapid development of new ports and harbours around the world, further robust data on the sediment deposition and turbidity tolerances of key organisms are required. These results highlight the role that controlled laboratory-based experiments can have in providing key inputs into the understanding of potential impacts of dredging-related activities on marine organisms and systems.

## Materials and Methods

### Study Species and Sampling Design

Two common Indo-Pacific corals were selected for the current study: the branching *Acropora millepora* and the foliaceous *Montipora aequituberculata*. These species represent two distinct morphologies that may be affected differently by sediment deposition and turbidity. Both species were collected from Viper Reef, in the Coral Sea (18°52.5′ S, 148°08.7′E) under permit No. G09/30237.1 (Great Barrier Reef Marine Park Authority). Six colonies per species were selected haphazardly at depths where these species occur in high abundance: 4–6 m for *A. millepora* and 12 m for *M. aequituberculata*. Coral colonies were maintained in 1000 l holding tanks with flow-through filtered seawater and 75% shading (approximately 200 µmol photons m^−2^ s^−1^) at the Australian Institute of Marine Science (AIMS) in Townsville.

Coral fragments, measuring approximately 5 cm, were obtained from each colony using surgical bone-cutters (*A. millepora*) or drilled using a 5-cm diameter diamond hole saw (*M. aequituberculata*). Fragments were then fixed to plastic blocks with underwater epoxy (Knead It, Selleys, Australia). Coral fragments were allowed to heal in outdoor holding tanks for at least 1 week. Afterwards, fragments were maintained in 30 l flow-through tanks (water temperature 26.4±0.9°C, salinity 33.2±2.6 ppt, photoperiod 12 h light:dark (214.6±1.2 µmol photons m^−2^ s^−1^), and acclimatized to laboratory conditions for another week prior to commencing the experiment.

### Experimental Facility

The indoor aquaria facilities at AIMS were used to deliver up to 100 mg l^−1^ of total suspended solids (TSS) to 18 treatment tanks using short frequent pulses of concentrated stock suspensions of sediments. The 240 l fiberglass concentrated stock tanks were custom made with a 45° taper at the base that eliminated the accumulation of sediments (Fig. 1). An external 2400 l h^−1^ pump (Eheim 1260: Eheim GmbH, Germany) was used to draw the water/sediment suspension from the base of each tank and deliver it back into the top of each tank, keeping sediments suspended. A second Eheim 1260 pump was used to pulse sediment suspensions (8 sec every 8 min, approx 120 ml) into the treatment tanks. The TSS was derived from daily turbidity readings taken from each experimental tank.

Fresh seawater was filtered to 0.5 µm and delivered to a header tank above the treatment tanks (Fig. 1). This header tank ensured constant pressure and was used to deliver 500 ml min^−1^ to each of the experimental tanks controlled by in-line flow meters and taps. The exposure of corals to the sediments was conducted in 18 white plastic tanks (30 l). A Eheim Compact+3000 pump (1500–3000 l h^−1^) was suspended at the waterline in the center of each experimental tank. This pump faced downwards and provided constant circulation and a flow over the corals of approximately 5–10 cm s^−1^, effectively maintaining the sediments in suspension (Fig. 1). Each treatment tank contained 9 fragments of each species and 3 fragments of each species were sampled at 4, 12 and 16 weeks from 3 replicate treatment tanks. Each tank was illuminated with four compact fluorescent tubes (420 nm, 55 W, Catalina) yielding an irradiance of approximately 200 µmol photons m^−2^ s^−1^. This irradiance is similar to the expected mean photosynthetically active radiation at the collection site. Water quality parameters were checked regularly and remained consistent throughout the duration of the experiment. Salinity, dissolved oxygen and turbidity were all measured using a 90-FLT water quality logger (TPS, Brisbane, Australia).

### Preparation of Sediments

To mimic the sediments produced within 500 m of dredging operations, fine sediments (mean particle size 6.4±0.8 (SE) µm, 95% <20 µm) were generated from offshore coral-derived sand and loose coral rubble. These sediments were ground in a 10 l ceramic ball mill with a charge of 5 kg of 20 mm ceramic balls (CSIRO Division of Minerals, Perth). The charge was recovered after milling and passed through a 150 µm sieve to remove any unground fragments such as shell or other organic material. The resulting slurry was then oven dried at 100°C before being stored in an air-tight container. Each milled charge resulted in approximately 3 kg of dried product. Approximately 50 kg dried sediments were used for the experiment.

Three random sediment samples (one from each of the original sediment containers) were taken for hydrocarbon, persistent organochlorine, butyltin, metal and nutrient analysis and the methods and results are tabulated in Tables S5, S6 and S7. Elemental analysis of the sediment sample confirmed that the samples were largely comprised of calcium carbonate (34% calcium and 12% carbon) (Tables S6 and S7). The total organic carbon and total nitrogen concentrations (Table S6) were consistent with those reported from coral reef sediments of the GBR [51]. Total phosphorus concentrations (Table S6) were also very similar to concentrations from the sediments of Davies Reef (GBR) [52]. The origins of the sediments in the present study are likely to be the skeletons of corals, crustose coralline algae and foraminifera.

Only low concentrations of hydrocarbons (TPH) were detected (<10 mg kg^−1^, data not shown), consistent with biogenic sources, including natural populations of bacteria and algae [53]. The persistent organic pollutants such as PAHs and PCBs, along with the antifoulant, tributyltin, were not detected in the sediment samples.

### Total Suspended Solids (TSS) and Light Attenuation

The sediment concentrations 0, 1, 3, 10, 30 and 100 mg l^−1^ TSS were maintained in suspension using an Eheim Compact+3000 pump and calibration measurements were conducted in three white plastic tanks (60 l). Turbidity (n = 14 replicates/treatment) was measured with a TPS 90-FLT water quality logger. Light attenuation (n = 12 replicates/treatment) was measured in the same plastic tanks as TSS by measuring photosynthetically active radiation (PAR) with a Li-250A light meter (Li-cor, Lincoln, NE, USA) at the height level of corals in the treatment tanks (15 cm below the surface). Several pilot trials over week-long periods were conducted to ensure sediment delivery, turbidity and sedimentation rates were consistent within tanks over time and between replicate tanks in a given treatment.

### Sediment Deposition Rate (SR)

Sediment deposition rates (sedimentation rates, SR) were measured weekly during the sediment dosing phase of the experiment and twice during recovery. Small sediment traps (20 ml glass vials, 15 mm opening diameter, 58 mm height) were secured in each experimental tank with the top at the height of the corals and remained deployed for 24 h. The contents of each trap were filtered through pre-weighed 0.4-µm polycarbonate filters. Filters were dried at 60°C until constant weight. Sedimentation rates (in mg cm^−2^ day^−1^) were calculated as per Equation 1:
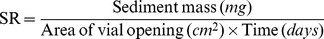
(1)


### Mortality and Sediment Accumulation

The percentage of partial mortality, full mortality and sediment deposition of *M. aequituberculata* and growth rates (extension) of *A. millepora* were measured via imaging process and analysis software, Image J (U.S. NIH, MD, USA http://rsb.info.nih.gov/ij/). A digital camera was fixed 27 cm above the coral to provide consistent height from each coral sample during photography. Images of *M. aequituberculata* (top view) and *A. millepora* (front and left views) were taken over a 5×5 cm grid. These images were then imported into Image J to calculate percent sediment deposition and partial mortality of *M. aequituberculata* and length of *A. millepora* from tip to base. The same light conditions, aperture and shutter speed were used to capture each image. An *A. millepora* image from t = 0 and the sampling day (either t = 12, 16 weeks) were opened simultaneously to ensure that measurements were performed using the same aspect. If the coral had uneven extension at the base, measurements were made from the halfway point of base extension to the tip of the coral. Sampling day (t = 4) was not used for extension measurements due to insufficient growth at that time point.

### Physiological and Biochemical Analyses

Tissue for biochemical analyses was obtained from coral fragments by stripping each sample with an air gun (Jamec Pem, Australia) and 0.22-µm filtered seawater (20–25 ml). The volume of this blastate was recorded and the blastate homogenized for 30 s (Heidolph GmbH, Germany). Aliquots were taken for: total proteins (1 ml); total lipids (10 ml); symbiont density (1 ml); and pigment analysis (1.5 ml). Aliquots for lipid and protein analyses were stored at −20°C while aliquots for symbiont density were immediately fixed in 10% buffered formalin. Aliquots for pigment analysis were immediately centrifuged at 1500×g for 5 min. The supernatant was discarded leaving a pellet containing the zooxanthellae then stored at −80°C for future pigment analysis.

Aliquots for lipid analysis (10 ml) were freeze dried and extracted twice with dichloromethane-methanol (2∶1 v/v). The slurry was filtered through a GF/C filter (Whatman) and washed in 0.88% KCl followed by three washes with methanol-H_2_O (1∶1 v/v) [54]. The lower dichloromethane phase was decanted into an aluminum pan of known weight, and the dichloromethane evaporated at 60°C overnight. Pans were allowed to cool until constant weight was reached. Lipids were normalized to biomass as using protein content as a proxy (lipid/protein) [55]. Total protein content in the 200 µl coral tissue aliquots was determined spectrophotometrically using the Bio-Rad DC protein assay kit (Richmond, CA, USA) with bovine serum albumin (BSA, Sigma-Aldrich) as a standard [56]. Absorbances were measured at 750 nm using a multi-detection microplate reader (Bio-Tek Instruments, Inc., Winooski, VT, USA). Measurements were made in duplicate and samples were re-run when coefficient of variation exceeded 10%.

Chlorophyll fluorescence of symbiotic dinoflagellate algae within live tissues of *A. millepora* (not covered with sediments) was measured using the Diving-PAM fluorometer (Walz GmbH, Germany). Measurements were obtained in the dark at least 5 mm from the tip of the coral and 3 mm from the coral tissue (controlled via a rubber spacer) by placing a 6 mm fibre-optic probe perpendicular to the surface of the coral. Initial fluorescence (*F_0_* in dark-adapted samples) was determined by applying a weak pulse-modulated red measuring light (650 nm, 0.15 µmol photons m^−2^s^−1^). The maximum fluorescence (Fm in dark-adapted samples) was then measured, following application of a saturating pulse of actinic light. Maximum quantum yield (F_v_/F_m_) is the proportion of light used for photosynthesis by chlorophyll when all reaction centers are open [57]. Corals were dark adapted for 30 min and maximum quantum yields were obtained per Equation 2:
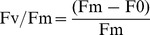
(2)Maximum quantum yield (F_v_/F_m_) values were conducted weekly from 3 randomly selected dark-acclimated corals per species from each treatment. F_v_/F_m_ measurements were taken only on live tissue of both species and between 1 and 4 measurements were taken from the tissue of each colony depending on the amount of live tissue available. We did not report PAM measurements from symbionts in buried coral tissue as endolithic algae would have dominated the fluorescence signal from these regions within colonies.

Chlorophyll *a* content was analysed as a proxy for coral bleaching (loss of symbiotic zooxanthellae). Chlorophyll *a* content in coral tissue homogenates was analyzed by using high performance liquid chromatography (HPLC) (Waters Corp., Milford, MA, USA) in combination with a Phenomonex C-18 Gemini 110Å column according to the method of Cantin et al. [58]. Chlorophyll *a* (chl *a*) was normalized to protein content as a proxy for biomass (see Lipid Content section above). Chlorophyll *a* was compared with zooxanthellae density for randomly selected colonies of *A. millepora* (r^2^ = 0.62, n  = 56, p<0.01) and *M. aequituberculata* (r^2^ = 0.64, n  = 50, p<0.01) across all treatments. These two indicators of coral bleaching were significantly correlated in both coral species and the cellular chl *a* concentrations of symbionts from each species were identical: 7.1±0.2 pg cell^−1^ and 6.9±0.3 pg cell^−1^ (SE) for *A. millepora* and *M. aequituberculata* respectively (t_104_ = 0.636, p  = 0.53).

### Statistical Analysis

Data were analyzed using one and two-way analysis of variance tests performed with Number Cruncher Statistical Software (NCSS) 2007 (Statistical and Power Analysis Software). Data were checked for normality and homogeneity of variances. Pooling procedures involving elimination of terms from the mean square estimates were implemented if a term was non-significant at *p*>0.25 [59]. Means for significant factors in the ANOVAs were compared using Tukey-Kramer comparison tests. Repeated measures ANOVA was used to assess differences in sedimentation rates over time. In contrast, the replicate level for all biochemical and physiological comparisons was n = 3 tank (with n = 3 corals of each species pooled per tank). Repeated measures ANOVAs were not applied to the analyses of the biochemical and physiological data since PAM fluorometry was performed on random corals within each treatment, and the destructive techniques required for lipid and chl *a* analyses were performed only once for a given coral fragment.

## Supporting Information

Figure S1 **Total suspended solids (TSS, mg l^−1^) in each of the experimental treatments over the exposure period. See** **Table 1** **for mean values.**
(TIF)

Figure S2 Light attenuation relative to control (0 mg l^−1^ TSS). Bars represent ± SE. * represents significantly different attenuation from 0 mg l^−1^ TSS (p<0.01).
(TIF)

Figure S3 **Sediment deposition rates (mg cm^−2^ day^−1^) in each of the experimental treatments over the exposure period. See** **Table 1** **for mean values.**
(TIF)

Figure S4 **The influence of total suspended solids on linear extension in** ***A. millepora*** **after 12 week sediment exposure plus a 4 week recovery.**
(TIF)

Table S1 **Results of ANOVA comparing sediment accumulation on the surface of Montipora aequituberculata between sampling times (t = 4 and 12 weeks) and sediment treatments.** Only the 4 most intense treatments were compared as no accumulation was observed in the lowest treatments.
(DOCX)

Table S2 **Summary of ANOVA of lipid content (mg cm^−2^) of Acropora millepora and Montipora aequituberculata normalized to protein (mg cm^−2^) between sampling times (t = 0, 4 and 12 weeks) and sediment treatments, and between termination of dosage (t = 12 weeks) compared to recovery (2 = 16 weeks).**
(DOCX)

Figure S3 **Summary of ANOVA comparing maximum quantum yield (Fv/Fm) of A. millepora and M. aequituberculata among sampling times (t = 0, 4 and 12 weeks) and sediment treatments, and yields at termination of experiment versus yields after recovery (t = 12 and 16weeks).**
(DOCX)

Table S4 **Summary of ANOVA comparing the ratio of chlorophyll a content to biomass of A. millepora and M. aequituberculata among sampling times (t = 0, 4 and 12 weeks) and sediment treatments, and chl a/biomass at termination of experiment versus after recovery (t = 12 and 16 weeks).**
(DOCX)

Table S5 **Analyses performed on the sediment samples used in the present experiments.**
(DOCX)

Table S6 **Analysis results for nutrients from 3 independent sediment samples.**
(DOCX)

Table S7 **Elemental analysis results from 3 independent sediment samples.**
(DOCX)
