# Supplementary figures and images for: Chronic Exposure of Corals to Fine Sediments: Lethal and Sub-Lethal Impacts
Source: PLoS One. 2012 May 25;7(5):e37795. doi: 10.1371/journal.pone.0037795 (PMC3360596; doi:10.1371/journal.pone.0037795)

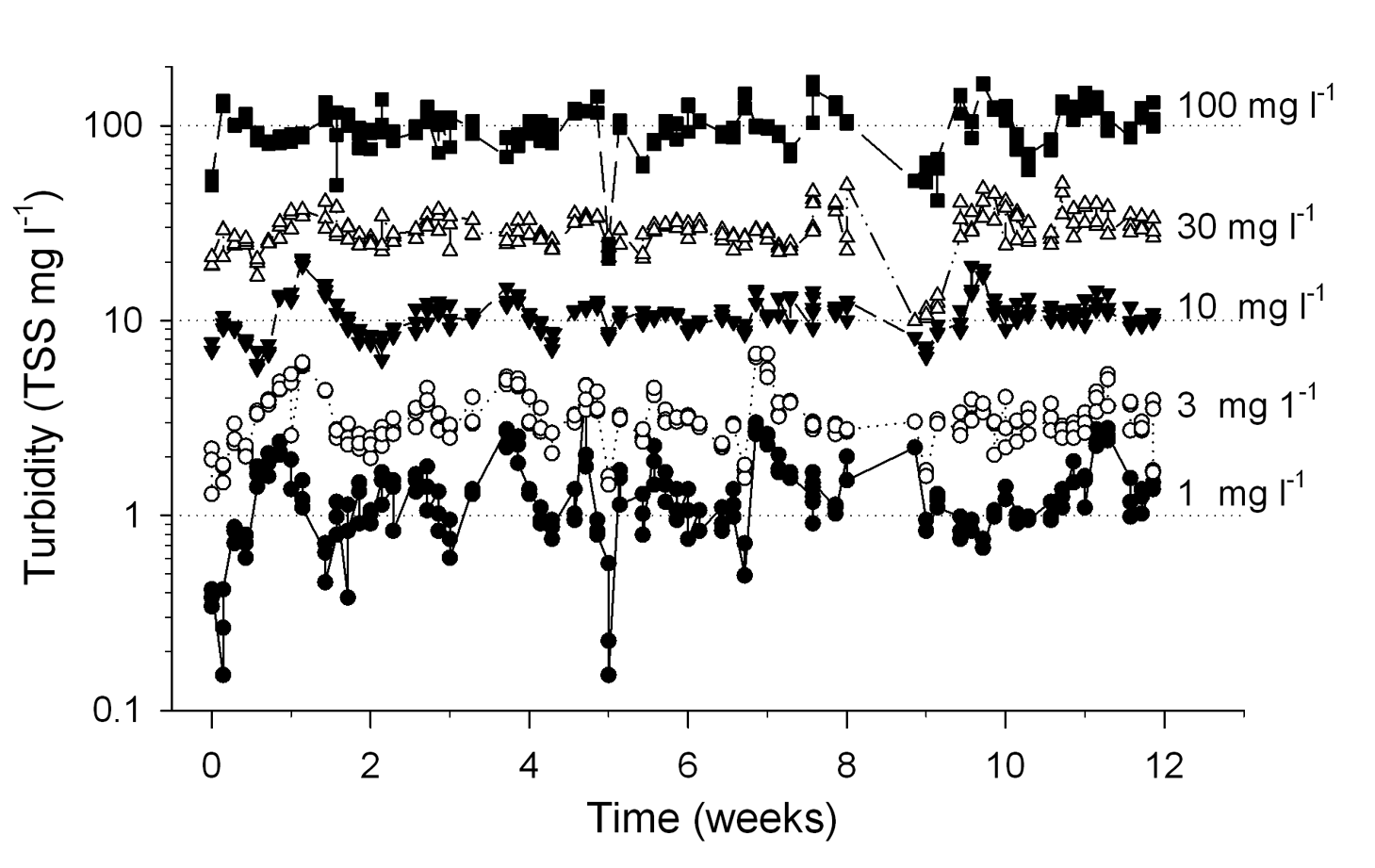

Supplement: Figure S1 — Total suspended solids (TSS, mg l−1) in each of the experimental treatments over the exposure period. See Table 1 for mean values. (TIF) [file pone.0037795.s001.tif]

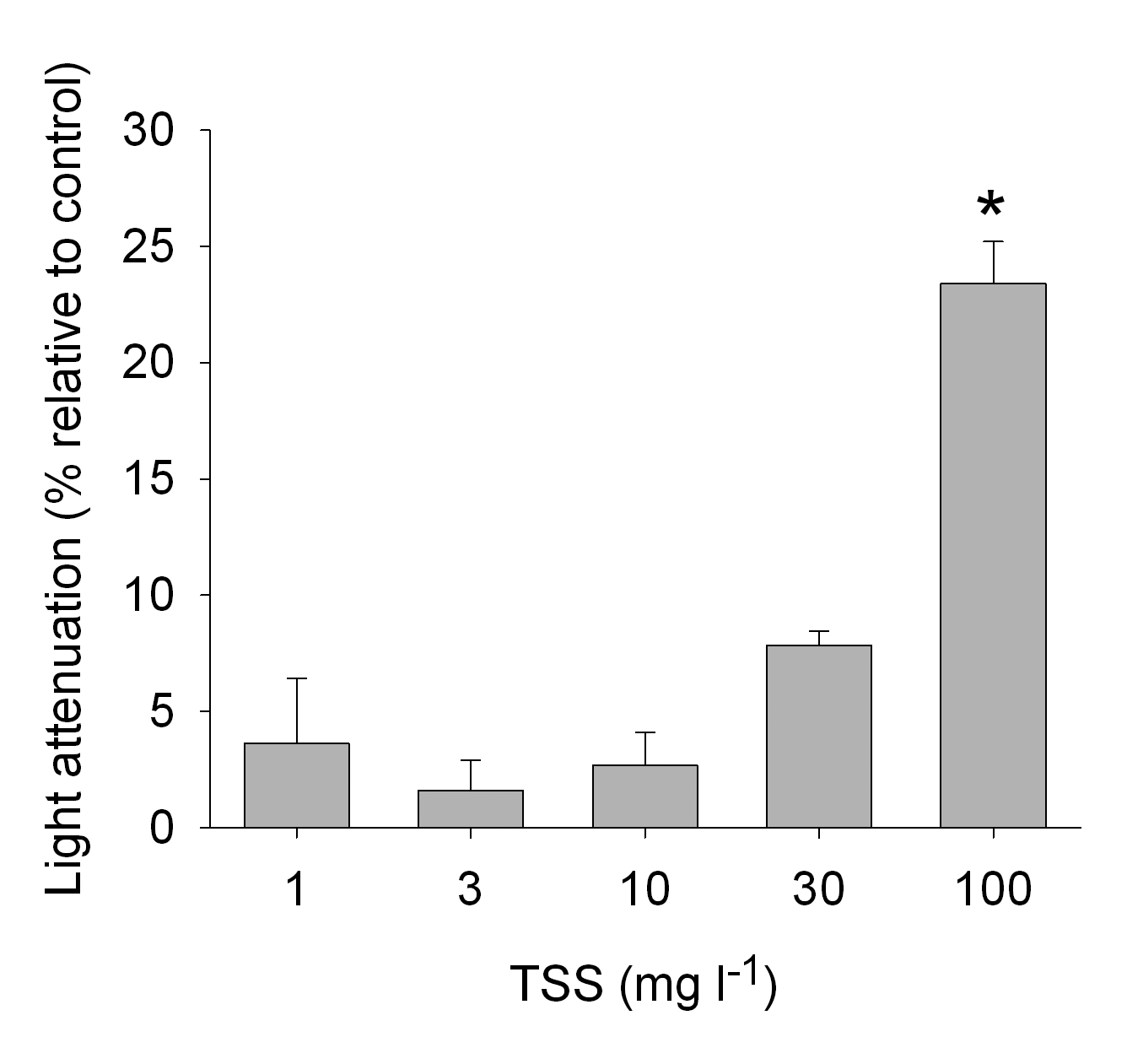

Supplement: Figure S2 — Light attenuation relative to control (0 mg l−1 TSS). Bars represent ± SE. * represents significantly different attenuation from 0 mg l−1 TSS (p<0.01). (TIF) [file pone.0037795.s002.tif]

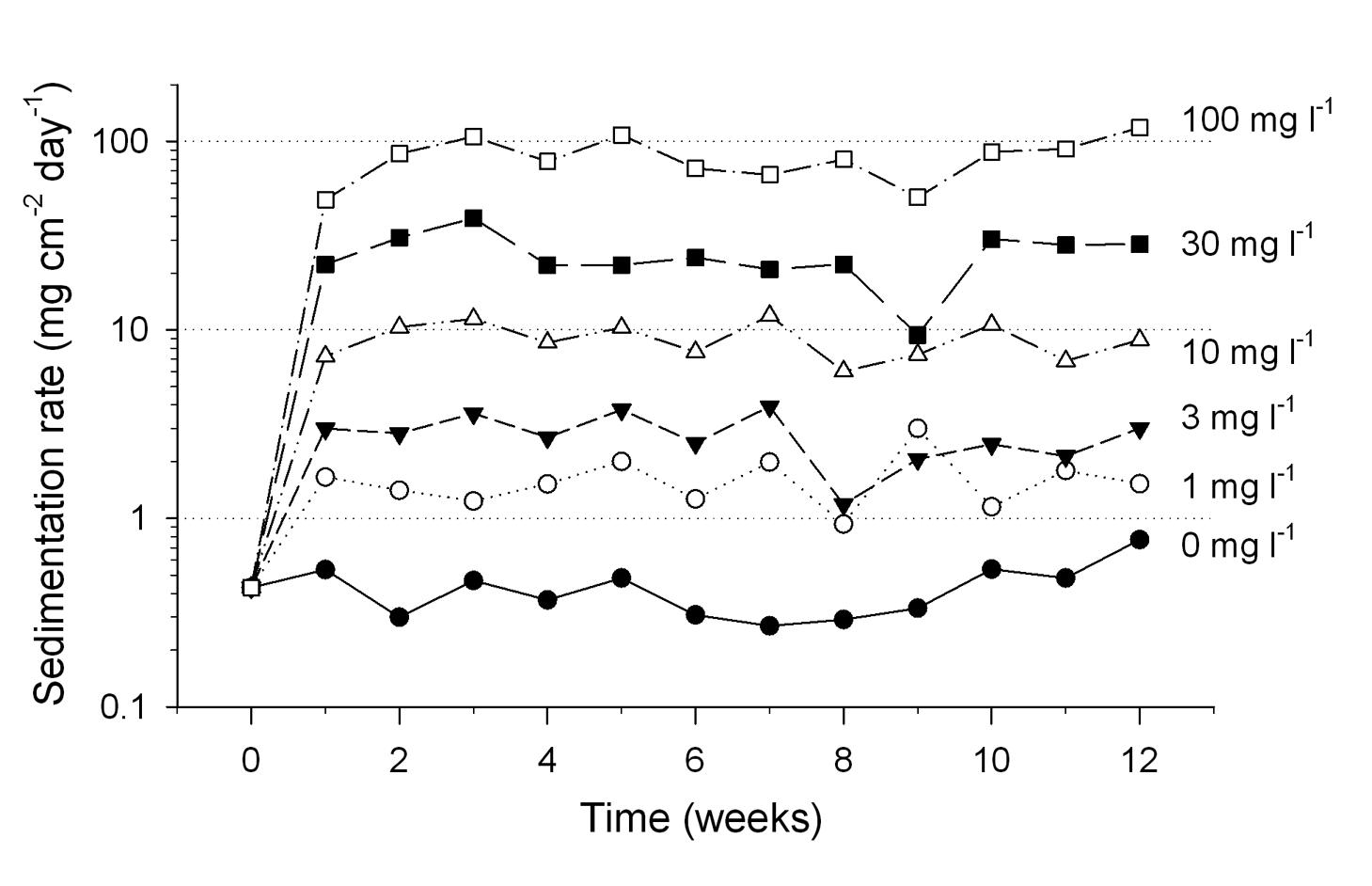

Supplement: Figure S3 — Sediment deposition rates (mg cm−2 day−1) in each of the experimental treatments over the exposure period. See Table 1 for mean values. (TIF) [file pone.0037795.s003.tif]

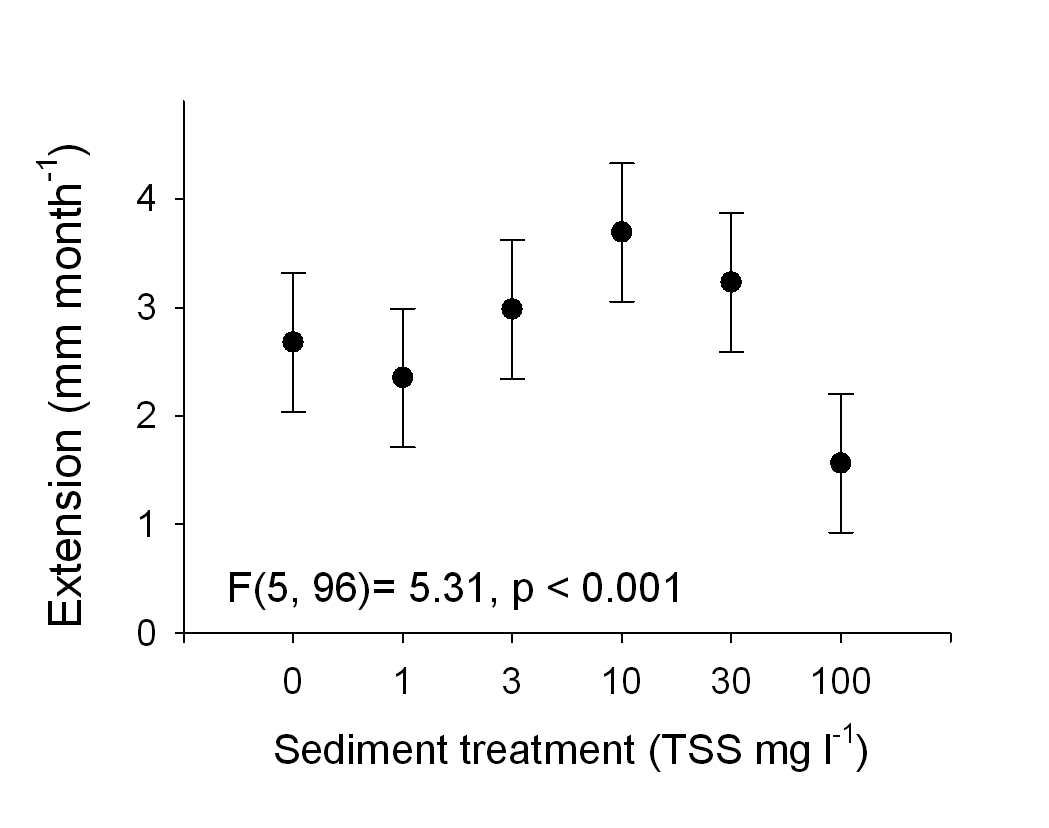

Supplement: Figure S4 — The influence of total suspended solids on linear extension in A. millepora after 12 week sediment exposure plus a 4 week recovery. (TIF) [file pone.0037795.s004.tif]
