# Supplementary material for: Chronic Exposure of Corals to Fine Sediments: Lethal and Sub-Lethal Impacts
Source: PLoS One. 2012 May 25;7(5):e37795. doi: 10.1371/journal.pone.0037795 (PMC3360596; doi:10.1371/journal.pone.0037795)
Supplement: Table S1 — Results of ANOVA comparing sediment accumulation on the surface of Montipora aequituberculata between sampling times (t = 4 and 12 weeks) and sediment treatments. Only the 4 most intense treatments were compared as no accumulation was observed in the lowest treatments. (DOCX) [file pone.0037795.s005.docx]

Table S1. Results of ANOVA comparing sediment accumulation on the surface of *Montipora aequituberculata* between sampling times (t = 4 and 12 weeks) and sediment treatments. Only the 4 most intense treatments were compared as no accumulation was observed in the lowest treatments.

| **Source** | **SS** | **df** | **MS** | **F** | **p** |
| --- | --- | --- | --- | --- | --- |
| Sampling time | 4016.31 | 1 | 4016.31 | 10.95 | 0.002* |
| TSS | 26093.8 | 3 | 8697.94 | 23.71 | < 0.001* |
| Time x TSS | 1100.90 | 3 | 366.97 | 1.00 | 0.404 |
| Error | 12842.11 | 35 | 366.92 |  |  |
