# Supplementary material for: Chronic Exposure of Corals to Fine Sediments: Lethal and Sub-Lethal Impacts
Source: PLoS One. 2012 May 25;7(5):e37795. doi: 10.1371/journal.pone.0037795 (PMC3360596; doi:10.1371/journal.pone.0037795)
Supplement: Table S2 — Summary of ANOVA of lipid content (mg cm−2) of Acropora millepora and Montipora aequituberculata normalized to protein (mg cm−2) between sampling times (t = 0, 4 and 12 weeks) and sediment treatments, and between termination of dosage (t = 12 weeks) compared to recovery (2 = 16 weeks). (DOCX) [file pone.0037795.s006.docx]

Table S2. Summary of ANOVA of lipid content (mg cm^-2^) of *Acropora millepora* and *Montipora aequituberculata* normalized to protein (mg cm^-2^) between sampling times (t = 0, 4 and 12 weeks) and sediment treatments, and between termination of dosage (t = 12 weeks) compared to recovery (2 = 16 weeks).

| **Source** | **SS** | **df** | **MS** | **F** | **p** |
| --- | --- | --- | --- | --- | --- |
| *A. millepora* |  |  |  |  |  |
| Exposure, t=0, 4, 12 wks |  |  |  |  |  |
| Sampling time | 7.89 x 10^-2^ | 2 | 3.94 x 10^-2^ | 50.0 | < 0.001* |
| TSS | 6.72 x 10^-3^ | 5 | 1.34 x 10^-3^ | 1.70 | 0.159 |
| Time x TSS | 8.50 x 10^-2^ | 10 | 8.50 x 10^-3^ | 10.78 | < 0.001* |
| Error | 2.84 x 10^-2^ | 36 | 7.89 x 10^-4^ |  |  |
| Recovery, 12 vs 16 wks |  |  |  |  |  |
| Sampling time | 1.54 x 10^-2^ | 1 | 1.54 x 10^-2^ | 6.65 | 0.017* |
| TSS | 1.44 x 10^-2^ | 5 | 2.89 x 10^-3^ | 1.24 | 0.321 |
| Time x TSS | 1.07 x 10^-2^ | 5 | 2.15 x 10^-2^ | 9.23 | < 0.001* |
| Error | 5.58 x 10^-2^ | 24 | 2.32 x 10^-3^ |  |  |
| *M. aequituberculata* |  |  |  |  |  |
| Exposure, t=0, 4, 12 wks |  |  |  |  |  |
| Sampling time | 0.12 | 2 | 5.80 x 10^-2^ | 24.53 | < 0.001* |
| TSS | 4.68 x 10^-2^ | 5 | 9.37 x 10^-3^ | 3.96 | 0.006* |
| Time x TSS | 7.32 x 10^-2^ | 10 | 7.32 x 10^-3^ | 3.09 | 0.007* |
| Error | 8.04 x 10^-2^ | 34 | 2.37 x 10^-3^ |  |  |
| Recovery, 12 vs 16 wks |  |  |  |  |  |
| Sampling time | 2.47 x 10^-2^ | 1 | 2.47 x 10^-2^ | 14.39 | 0.001* |
| TSS | 1.84 x 10^-2^ | 5 | 3.67 x 10^-3^ | 2.14 | 0.099 |
| Time x TSS | 9.79 x 10^-3^ | 5 | 1.96 x 10^-3^ | 1.14 | 0.369 |
| Error | 3.78 x 10^-2^ | 22 | 1.72 x 10^-3^ |  |  |
