# Supplementary material for: Chronic Exposure of Corals to Fine Sediments: Lethal and Sub-Lethal Impacts
Source: PLoS One. 2012 May 25;7(5):e37795. doi: 10.1371/journal.pone.0037795 (PMC3360596; doi:10.1371/journal.pone.0037795)
Supplement: Figure S3 — Summary of ANOVA comparing maximum quantum yield (Fv/Fm) of A. millepora and M. aequituberculata among sampling times (t = 0, 4 and 12 weeks) and sediment treatments, and yields at termination of experiment versus yields after recovery (t = 12 and 16weeks). (DOCX) [file pone.0037795.s007.docx]

Table S3. Summary of ANOVA comparing maximum quantum yield (F_v_/F_m_) of *A. millepora* and *M. aequituberculata* among sampling times (t = 0, 4 and 12 weeks) and sediment treatments, and yields at termination of experiment versus yields after recovery (t = 12 and 16weeks).

| **Source** | **SS** | **df** | **MS** | **F** | **P** |
| --- | --- | --- | --- | --- | --- |
| *A. millepora* |  |  |  |  |  |
| Exposure, t=0, 4, 12 wks |  |  |  |  |  |
| Sampling time | 3.05 x 10^-2^ | 2 | 1.52 x 10^-2^ | 151.14 | < 0.001* |
| TSS | 9.97 | 5 | 1.99 x 10^-4^ | 1.98 | 0.106 |
| Time x TSS | 6.89 x 10^-3^ | 10 | 6.89 x 10^-4^ | 6.84 | < 0.001* |
| Error | 3.63 x 10^-3^ | 36 | 1.01 x 10^-4^ |  |  |
| Recovery, 12 vs 16 wks |  |  |  |  |  |
| Sampling time | 4.02 x 10^-3^ | 1 | 4.02 x 10^-3^ | 19.74 | < 0.001* |
| TSS | 9.09 x 10^-3^ | 5 | 1.82 x 10^-3^ | 8.93 | < 0.001* |
| Time x TSS | 1.07 x 10^-3^ | 5 | 2.13 x 10^-4^ | 1.05 | 0.414 |
| Error | 4.89 x 10^-3^ | 24 | 2.04 x 10^-4^ |  |  |
| *M. aequituberculata* |  |  |  |  |  |
| Exposure, t=0, 4, 12 wks |  |  |  |  |  |
| Sampling time | 0.19 | 2 | 9.37 x 10^-2^ | 102.80 | < 0.001* |
| TSS | 1.44 | 5 | 2.89 x 10^-3^ | 3.17 | 0.018* |
| Time x TSS | 1.42 x 10^-2^ | 10 | 1.42 x 10^-3^ | 1.56 | 0.159 |
| Error | 3.28 | 36 | 9.11 x 10^-4^ |  |  |
| Recovery, 12 vs 16 wks |  |  |  |  |  |
| Sampling time | 5.88 x 10^-3^ | 1 | 5.88 x 10^-3^ | 3.24 | 0.084 |
| TSS | 4.74 x 10^-2^ | 5 | 9.49 x 10^-3^ | 5.23 | 0.002* |
| Time x TSS | 2.13 x 10^-4^ | 5 | 4.27 x 10^-5^ | 0.02 | 1.000 |
| Error | 4.36 x 10^-2^ | 24 | 1.82 x 10^-3^ |  |  |
