# Supplementary material for: Chronic Exposure of Corals to Fine Sediments: Lethal and Sub-Lethal Impacts
Source: PLoS One. 2012 May 25;7(5):e37795. doi: 10.1371/journal.pone.0037795 (PMC3360596; doi:10.1371/journal.pone.0037795)
Supplement: Table S4 — Summary of ANOVA comparing the ratio of chlorophyll a content to biomass of A. millepora and M. aequituberculata among sampling times (t = 0, 4 and 12 weeks) and sediment treatments, and chl a/biomass at termination of experiment versus after recovery (t = 12 and 16 weeks). (DOCX) [file pone.0037795.s008.docx]

Table S4. Summary of ANOVA comparing the ratio of chlorophyll *a* content to biomass of *A. millepora* and *M. aequituberculata* among sampling times (t = 0, 4 and 12 weeks) and sediment treatments, and chl *a/*biomass at termination of experiment versus after recovery (t = 12 and 16 weeks).

| **Source** | **SS** | **df** | **MS** | **F** | **p** |
| --- | --- | --- | --- | --- | --- |
| *A. millepora* |  |  |  |  |  |
| Exposure, t=0, 4, 12 wks |  |  |  |  |  |
| Sampling time | 2.29 x 10^-4^ | 2 | 1.15 x 10^-4^ | 145.01 | < 0.001* |
| TSS | 2.54 x 10^-5^ | 5 | 5.09 x 10^-6^ | 6.44 | < 0.001* |
| Time x TSS | 2.11 x 10^-5^ | 10 | 2.11 x 10^-6^ | 2.67 | 0.015* |
| Error | 4.71 x 10^-4^ | 36 | 3.32 x 10^-6^ |  |  |
| Recovery, 12 vs 16 wks |  |  |  |  |  |
| Sampling time | 1.78 x 10^-5^ | 1 | 1.78 x 10^-5^ | 15.57 | 0.001* |
| TSS | 2.64 x 10^-5^ | 5 | 5.28 x 10^-6^ | 4.61 | 0.004* |
| Time x TSS | 7.25 x 10^-6^ | 5 | 1.45 x 10^-6^ | 1.27 | 0.310 |
| Error | 2.75 x 10^-5^ | 24 | 1.15 x 10^-6^ |  |  |
| *M. aequituberculata* |  |  |  |  |  |
| Exposure, t=0, 4, 12 wks |  |  |  |  |  |
| Sampling time | 5.01 x 10^-5^ | 2 | 2.51 x 10^-5^ | 11.46 | < 0.001* |
| TSS | 9.13 x 10^-6^ | 5 | 1.83 x 10^-6^ | 0.83 | 0.534 |
| Time x TSS | 9.55 x 10^-6^ | 10 | 9.55 x 10^-7^ | 0.44 | 0.918 |
| Error | 7.87 x 10^-5^ | 36 | 2.19 x 10^-6^ |  |  |
| Recovery, 12 vs 16 wks |  |  |  |  |  |
| Sampling time | 1.76 x 10^-8^ | 1 | 1.760 x 10^-8^ | 0.01 | 0.926 |
| TSS | 4.71 x 10^-5^ | 5 | 9.43 x 10^-7^ | 0.47 | 0.795 |
| Time x TSS | 1.99 x 10^-5^ | 5 | 3.99 x 10^-6^ | 1.99 | 0.117 |
| Error | 4.82 x 10^-5^ | 24 | 2.01 x 10^-6^ |  |  |
