# Supplementary material for: Chronic Exposure of Corals to Fine Sediments: Lethal and Sub-Lethal Impacts
Source: PLoS One. 2012 May 25;7(5):e37795. doi: 10.1371/journal.pone.0037795 (PMC3360596; doi:10.1371/journal.pone.0037795)
Supplement: Table S5 — Analyses performed on the sediment samples used in the present experiments. (DOCX) [file pone.0037795.s009.docx]

Table S5. Analyses performed on the sediment samples used in the present experiments.

| **Analyte** | **Analysis type** | **Provider** |
| --- | --- | --- |
| Total Nitrogen | Kjel | QHFSS^1^ |
| Total Phosphorus | ICPAES^3^ | QHFSS^1^ |
| Total Carbon | ICPAES^3^ | QHFSS^1^ |
| Total Organic Carbon | ICPAES^3^ | QHFSS^1^ |
| Elemental analysis (incuding metals) | ICPAES^3^ | QHFSS^1^ |
| Polycyclic aromatic hydrocarbons (PAH) | GC-MS^4^ | QHFSS^1^ |
| Polychlorinated biphenyls (PCB) | GC-MS^4^ | QHFSS^1^ |
| Total petroleum hydrocarbons (TPH) | GC-MS^4^ | QHFSS^1^ |
| Tributyltin (TBT) | GC-QFAAS^5^ | ALS^2^ |

^1^QHFSS = Queensland Health Forensic and Scientific Services (Archerfield, QLD)

^2^ALS = Laboratory Services Environmental Division (Brisbane, QLD)

^3^ICPAES = inductively coupled plasma-atomic emission spectrometry

^4^GC-MS = gas chromatography – mass spectrometry

^5^GC-QFAAS = gas chromatography––quartz furnace atomic absorption spectrometry
