# Supplementary material for: Chronic Exposure of Corals to Fine Sediments: Lethal and Sub-Lethal Impacts
Source: PLoS One. 2012 May 25;7(5):e37795. doi: 10.1371/journal.pone.0037795 (PMC3360596; doi:10.1371/journal.pone.0037795)
Supplement: Table S6 — Analysis results for nutrients from 3 independent sediment samples. (DOCX) [file pone.0037795.s010.docx]

Table S6. Analysis results for nutrients from 3 independent sediment samples.

| **Nutrient** | **Units** | **B1-M** | **B2-M** | **B3-M** |
| --- | --- | --- | --- | --- |
| Phosphorus | mg kg^-1^ | 280 | 290 | 290 |
| Nitrogen oxides | mg kg^-1^  as N | <0.1 | 0.1 | 0.2 |
| Total Kjel nitrogen | mg kg^-1^  as N | 540 | 490 | 490 |
| Total Carbon (% C, w/w) | % C, w/w | 11.6 | 11.6 | 11.5 |
| Total Organic Carbon (% C) | % C, w/w | 0.11 | 0.11 | 0.11 |
