# Supplementary material for: Chronic Exposure of Corals to Fine Sediments: Lethal and Sub-Lethal Impacts
Source: PLoS One. 2012 May 25;7(5):e37795. doi: 10.1371/journal.pone.0037795 (PMC3360596; doi:10.1371/journal.pone.0037795)
Supplement: Table S7 — Elemental analysis results from 3 independent sediment samples. (DOCX) [file pone.0037795.s011.docx]

Table S7. Elemental analysis results from 3 independent sediment samples.

| **Sample** | **B1-M** | **B2-M** | **B3-M** |
| --- | --- | --- | --- |
| **Elements** | **mg kg^-1^** | **mg kg^-1^** | **mg kg^-1^** |
| Aluminium | 82 | 99 | 110 |
| Antimony | <70 | <70 | <70 |
| Arsenic | <40 | <40 | <40 |
| Barium | 16 | 17 | 21 |
| Beryllium | <20 | <20 | <20 |
| Calcium | 336,400 | 340,600 | 338,200 |
| Cadmium | <4 | <4 | <4 |
| Chromium | <4 | <4 | <4 |
| Cobalt | <5 | <5 | <5 |
| Copper | <30 | <30 | <30 |
| Iron | 51 | 56 | 62 |
| Potassium | 190 | 140 | 97 |
| Lead | <10 | <10 | <10 |
| Magnesium | 14,000 | 14,100 | 14,000 |
| Manganese | 6.9 | 6.9 | 6.8 |
| Mercury | <10 | <10 | <10 |
| Molybdenum | <5 | <5 | <5 |
| Nickel | <5 | <5 | <5 |
| Selenium | <40 | <40 | <40 |
| Sodium | 10,100 | 8600 | 7900 |
| Sulphur | 2600 | 2400 | 2200 |
| Tin | <20 | <20 | <20 |
| Vanadium | <3 | <3 | <3 |
| Zinc | <4 | <4 | <4 |
